# Curcumin Protects Skin against UVB-Induced Cytotoxicity via the Keap1-Nrf2 Pathway: The Use of a Microemulsion Delivery System

**DOI:** 10.1155/2017/5205471

**Published:** 2017-07-05

**Authors:** Maya Ben Yehuda Greenwald, Marina Frušić-Zlotkin, Yoram Soroka, Shmuel Ben Sasson, Ronit Bitton, Havazelet Bianco-Peled, Ron Kohen

**Affiliations:** ^1^The Institute for Drug Research, School of Pharmacy, The Hebrew University of Jerusalem, 9112100 Jerusalem, Israel; ^2^Department of Chemical Engineering, Technion-Israel Institute of Technology, Technion City, 3200003 Haifa, Israel; ^3^The Russell Berrie Nanotechnology Institute and Technion-Israel Institute of Technology, 32000 Haifa, Israel; ^4^Department of Developmental Biology and Cancer Research, The Hebrew University Medical School, Ein-Karem Campus, 9112100 Jerusalem, Israel; ^5^Department of Chemical Engineering, Ben-Gurion University of the Negev, 8410501 Beer-Sheva, Israel; ^6^Ilse Katz Institute for Nanoscale Science and Technology, 8410501 Beer-Sheva, Israel

## Abstract

Curcumin was found to be beneficial in treating several skin pathologies and diseases, providing antioxidant protection due to its reducing properties and its electrophilic properties (the ability to activate the Nrf_2_ pathway and induce phase II cytoprotective enzymes). Nevertheless, clinical applications of curcumin are being hampered by its insufficient solubility, chemical instability, and poor absorption, leading to low efficacy in preventing skin pathologies. These limitations can be overcome by using a nanotechnology-based delivery system. Here, we elucidated the possibility of using curcumin encapsulated in a microemulsion preserving its unique chemical structure. We also examined whether curcumin microemulsion would reduce UVB-induced toxicity in skin. A significant curcumin concentration was found in the human skin dermis following topical application of a curcumin microemulsion. Moreover, curcumin microemulsion enhanced the reduction of UV-induced cytotoxicity in epidermal cells, paving the way for other incorporated electrophiles in encapsulated form protecting skin against stress-related diseases.

## 1. Introduction

The concept that antioxidants can protect cells and organs against oxidative stress has been established in numerous basic and clinical studies [1]. Nevertheless, nowadays, it has become evident that antioxidants of low molecular weight cannot protect the living organism against continuous stress and sometimes can even be deleterious [2]. Oxidants (electrophiles), on the other hand, were recently shown to be compounds capable of inducing cellular-protecting enzymes such as the phase II enzymes when provided in moderate concentrations. One of the basic factors activated when an electrophile is present is the transcription factor nuclear factor (erythroid-derived 2)-like 2, an NF-E2-related factor 2 (Nrf2), which is responsible for the induction of a variety of cytoprotective genes [3]. Regulated by the Keap1 metalloprotein, Nrf2 is capable of inducing a large number of genes encoding antioxidant enzymes and genes enabling homeostasis and controlling processes involved in the pathology of many diseases (e.g., immune and inflammatory responses, tissue remodeling and fibrosis, carcinogenesis, and metastasis) [4, 5]. Nrf2 plays a vital and crucial role in the maintenance of skin homeostasis and repair and regeneration in various disease states of the skin [6]. However, acute and chronic Nrf2 activation in a healthy epidermis resulted in a negative effect on skin integrity [6]. Endogenous Nrf2 has the ability to protect skin against UV irradiation [6]. Nrf2 is also capable of decreasing symptoms of skin photoaging (e.g., wrinkle formation, loss of skin flexibility) [6]. The pharmacological activation of Nrf2 was proven to provide protection against various toxic compounds responsible for a reduction in skin toxicity [6]. The role of Nrf2 in the prevention of skin carcinogenesis has been demonstrated in various research models [6]. Nrf2 is a key element in the prevention of chemically induced tumor formation and promotion [6]. Moreover, Nrf2 activation reduced solar-simulated UV radiation tumor formation in hairless mice [6]. Nrf2 also demonstrated its essentiality in the healing process of full-thickness wounds and in the recovery and repair of an epidermal barrier defect [6]. There are compelling evidences demonstrating Nrf2 activation as a promising strategy for the treatment of atopic dermatitis, psoriasis, and epidermal blistering diseases (e.g., Hailey-Hailey disease) [6]. Nrf2 activation in vitiligo vulgaris pigment disorder was investigated as a potential strategy to prevent the development of the disorder and treatment [6]. It was also suggested that activation of Nrf2 is important for the treatment of patients suffering from allergic skin inflammation (e.g., allergic contact dermatitis) [6].

Curcumin (1,7-bis(4-hydroxy 3-methoxy phenyl)-1,6-heptadiene-3,5-dione) is a natural polyphenol from the powdered rhizome of the medicinal plant *Curcuma longa* (also known as turmeric) [7]. It is an amphipathic molecule with polar-central and flanking regions that are separated by a lipophilic methine segment [8]. Curcumin contains seven chemical functional groups (see curcumin chemical structure in the Supplementary Data (Figure S1) available online at https://doi.org/10.1155/2017/5205471) [8]. Among others, curcumin contains phenolic groups and thus can act as a reducing antioxidant and directly scavenge oxygen-centered reactive intermediates [8, 9]. Curcumin also displays oxidant activity partly due to its Michael acceptor functionalities. As such, curcumin is capable of inducing the activation of the Keap1-Nrf_2_-EpRE pathway [9]. The unique chemical attributes of curcumin (e.g., log *P* ensuring curcumin's accessibility to its molecular targets, the capacity to undergo H-bonding and hydrophobic interactions, and activity as a Michael acceptor) are responsible for curcumin's pleiotropic biological activity [8]. These include curcumin's bifunctional antioxidant properties, anti-inflammatory activity, anticancer effects, wound healing, and antimicrobial effects [8–14]. Therefore, curcumin was suggested for the treatment of various disorders like cancer and proinflammatory chronic diseases [8–14]. Skin, being an interface between the environment and the body, suffers from chronic oxidative stress resulting from exposure to environmental toxicants including chemical and physical pollutants, ionization, and UV radiation [15]. The resulting oxidative stress in skin may be involved in the pathogenesis of a number of skin disorders including some types of cutaneous malignancy and photosensitivity diseases [15]. Curcumin, due to its pleiotropic behavior, was found to be beneficial in treating several skin pathology disorders and diseases (e.g., psoriasis, scleroderma, and skin cancer) [8–14, 16–19]. Moreover, drug development studies were carried out where curcumin analogues were designed and synthesized due to curcumin's antiangiogenic activities [13]. The role of curcumin in treating various skin pathologies and disorders and Nrf2 involvement is summarized in Table 1.

However, the pharmacokinetics of curcumin are unsatisfactory due to its chemical instability, scarce solubility in aqueous solutions, deficient absorption, rapid metabolism, and systemic elimination [8, 20]. Therefore, curcumin suffers from poor bioavailability and its clinical application is restricted [8, 20]. Moreover, no double-blinded, placebo-controlled clinical trial of curcumin has been successful [21]. A reasonable approach to overcome these limitations could be to encapsulate curcumin into delivery systems of different characteristics [22, 23]. In addition, a topical delivery system for local administration of curcumin mat results in an increase in curcumin bioavailability [21]. There are compelling evidences supporting this approach. It was shown that topical application of curcumin exhibited a more pronounced effect on wound healing compared to its oral administration due to a superior accessibility of curcumin at the wound site [10]. One of the leading vehicles for dermal drug delivery is microemulsions [24]. Microemulsions are isotropic colloidal nano-formulations, composed of water, oil, and surfactants [25]. These vehicles are thermodynamically stable and form almost spontaneously (without any energy input) to a transparent or slightly opalescent formulation of low viscosity [25]. The use of microemulsions offers many advantages including enhancement of drug solubility, protection of labile drugs, controlled drug release, augmentation in the rate and extent of absorption, and a decrease in patient side effects [24]. In addition, it has been shown that microemulsions significantly increase bioavailability compared with classical delivery systems such as emulsions, gels, and solutions [24].

Incorporating curcumin into a microemulsion may improve its water solubility and bioavailability and hence lead to better efficacy [21]. Until now, little work has focused on topical microemulsion delivery systems containing curcumin aimed at treating skin conditions [26–29]. Nano-formulations of curcumin might potentially improve the infiltration of curcumin into cutaneous cells [10]. Indeed, studies to date support this claim [26–29]; Lin et al. developed curcumin encapsulated in an oil-in-water microemulsion system and investigated its phase diagram and stability [29]. In vitro skin permeation assays have demonstrated time-dependent increases in permeated curcumin in stable microemulsion formulations. Enhanced skin permeability of curcumin encapsulated in microemulsions was also reported by Liu and Chang [28]. The vehicle composition significantly influenced curcumin solubility and skin permeability [28]. Teichmann et al. incorporated curcumin in an oil-in-water microemulsion and in an amphiphilic cream [30]. A deeper part of the stratum corneum was accessible, and significantly smaller amounts of curcumin were found on the skin surface following microemulsion application. Furthermore, curcumin was detected in hair follicles, indicating that the microemulsion penetrated into the complete follicular infundibula [30]. Liu and Huang demonstrated that the antimicrobial activity of curcumin-loaded myristic acid microemulsions against the skin pathogen *Staphylococcus epidermidis* was 12 times higher than that of curcumin dissolved in dimethyl sulfoxide (DMSO) [26]. Yutani et al. assessed the distribution of polyphenols in skin from a di-2-ethylhexyl sodium sulfosuccinate (Aerosol OT) microemulsion and detected enhanced intradermal delivery [27].

In the present study, we hypothesize that incorporating curcumin into a topical microemulsion delivery system will preserve its unique chemical structure allowing it to induce the Keap1-Nrf_2_-EpRE pathway more efficiently than the unformulated curcumin. This hypothesis holds an additional rationale; it was shown that chronic and enhanced activation of the Keap1-Nrf_2_-EpRE pathway in the epidermis suffers from several detrimental complications including defects in the epidermal barrier, inflammation, and induced keratinocyte hyperproliferation [31]. Thus, precise and temporary activation of the Keap1-Nrf_2_-EpRE in skin is essential [32].

Here, we suggest to expand our prior work demonstrating the feasibility of encapsulating Nrf2-activating agent into a delivery system. We have previously shown that three members of the nitroxide family representing synthetic stable radicals were encapsulated into a microemulsion delivery system resulting in enhanced Nrf2 activation, protection against UVB-induced injury, and relief in inflamed skin condition [33]. While encapsulating these synthetic antioxidants with diverse lipophilicity and ability to shuttle between the nitroxide radical, the reduced hydroxylamine, and the oxidized oxoammonium cation formed by one- and two-electron transfer reactions [33] (i.e., members of the nitroxide family) may be challenging, the case of encapsulating the natural polyphenol curcumin into a microemulsion delivery system holds different challenges since curcumin is prone to oxidative degradation and has low solubility in aqueous solution [8, 20].

In order to investigate our hypothesis, curcumin was incorporated into a microemulsion delivery system and microemulsion nanometric structure was evaluated using dynamic light scattering (DLS), small-angle light scattering (SAXS), and cryo-transmission electron microscopy (cryo-TEM) measurements. Moreover, the antioxidant activity of curcumin incorporated into the microemulsion was evaluated in vitro using oxygen radical absorbance capacity assay (ORAC), luminol-dependent chemiluminescence (LDCL) assay, and 2-diphenyl-lpicrylhydrazyl (DPPH) radical assay. The capability of a curcumin-loaded microemulsion to induce the Keap1-Nrf2-EpRE pathway was evaluated in keratinocyte culture and in human skin. Human skin organ culture was used to access the reduced UVB-induced cytotoxicity resulting from topical application of the curcumin-loaded microemulsion.

## 2. Material and Methods

^∗^Similar material and methods were used in [33].

### 2.1. Microemulsion Preparation

Microemulsions were prepared by first mixing the surfactants lauric acid (pKa = 5.3 at room temperature, Sigma-Aldrich, Israel), Span® 20 (sorbitan laurate, Sigma-Aldrich, Israel), and Tween® 80 (polysorbate 80, Sigma-Aldrich, Israel) with isopropyl myristate (IPM, Sigma-Aldrich, Israel). Upon receiving a transparent blend of surfactants and oil, curcumin (Sigma-Aldrich, Israel) was added to the solution and then mixed until completely dissolved. This step was followed by a drop-wise addition of double-distilled water (DDW; pH = 6.8 ± 0.2). Solutions were allowed to equilibrate for 24 h to obtain a clear oil-in-water microemulsion. The ratio of Tween 80® : Span 20 : lauric acid : IPM : curcumin was 33.3 : 1.6 : 1 : 5 : 1.3 and kept constant throughout the study. The final concentrations (%*w*/*w*) in the microemulsion were 26.8 : 1.3 : 0.8 : 4 : 1 : 66.1 for Tween 80, Span 20, lauric acid, isopropyl myristate, curcumin, and water, respectively. tert-Butylhydroquinone (tBHQ) and trolox were purchased from Sigma-Aldrich, Israel.

### 2.2. Dynamic Light Scattering (DLS)

DLS measurements on microemulsions (microemulsions were diluted to 1 : 100 with DDW; the final curcumin concentration was 0.01% *w*/*w* (0.27 mM)) were performed using a Zetasizer Nano Series (Malvern) and analyzed using Zetasizer software. The droplet diameter was calculated from the diffusion coefficient, using Stokes-Einstein equation [34].

### 2.3. Cryogenic Transmission Electron Microscopy (Cryo-TEM)

Cryo-TEM specimens were prepared in a controlled environment box using a vitrification robot (Vitrobot). 60 *μ*L of the microemulsion (the final curcumin concentration was 1% *w*/*w* (27.1 mM)) was dropped onto a glow-discharged TEM grid (300-mesh Cu Lacey substrate; Ted Pella Ltd.). Excess was automatically blotted with a filter paper, and the specimen was rapidly plunged into liquid ethane and transferred to liquid nitrogen where it was kept until used. Specimens were analyzed below −175°C using an FEI Tecnai 12G^2^ TWIN TEM operated at 120 kV in a low-dose mode and with a few micrometers under focus to increase phase contrast. Images were recorded with a Gatan charge-coupled device camera (model 794) and examined using Digital Micrograph software, Version 3.1.

### 2.4. Small-Angle X-ray Scattering (SAXS)

SAXS experiments were performed on microemulsions without further manipulations (the final curcumin concentration was 1% *w*/*w* (27.1 mM)) using a small-angle diffractometer (a Molecular Metrology SAXS system with Cu K*α* radiation from a sealed microfocus tube (MicroMax-002+S), two Göbel mirrors, and three-pinhole slits; the generator was powered at 45 kV and 0.9 mA). Scattering patterns were recorded by a 20 × 20 cm two-dimensional position-sensitive wire detector (gas-filled proportional type of Gabriel design with 200 *μ*m resolution) that was positioned 150 cm behind the sample. Scattered intensity *I* (*q*) was recorded in the interval 0.07 < *q* < 2.7 nm^−1^, where *q* is the scattering vector defined as *q* = (4*π*/*λ*) sin (*Ѳ*), where 2*Ѳ* is the scattering angle and *λ* is the radiation wavelength (0.1542 nm). Microemulsions were sealed in a thin-walled capillary (glass) of about 2 mm diameter and 0.01 mm wall thickness. Experiments were performed under vacuum at ambient temperature. Scattering curves were adjusted for counting time and sample absorption.

### 2.5. Spectrofluorometer Measurements

Curcumin location in the microemulsion was investigated using the fluorescent probe method [35], which can sense the microenvironment of the probe from changes in the intensity and wavelength of the emission peak. Curcumin's emission properties highly depend on its specific microenvironment; therefore, curcumin could be used directly as a probe [8, 36]. Curcumin was dissolved in different microemulsion components to a final concentration of 0.007% *w*/*w* (1.9 *μ*M), and fluorescence measurements were obtained using a Jobin Yvon Horiba Fluoromax 4 spectrofluorometer. The excitation source was a xenon arc lamp. The excitation and emission slit widths were 5 nm. Excitation was set at 450 nm, and emission was scanned from 460 nm to 600 nm.

### 2.6. Voltammetric Measurements of Reducing Power

The overall reducing power of microemulsions (the final curcumin concentrations were as follows (%*w*/*w*): 0 (empty microemulsion), 0.25 (6.8 mM), 0.5 (13.9 mM), 0.75 (20.4 mM), and 1 (27.1 mM)) was examined using a cyclic voltammeter (Electrochemical Analyzer, CH Instruments, Austin, TX, USA). Samples were placed in a well with three electrodes: a glassy carbon, with a working electrode of 3.3 mm diameter; an Ag/AgCl reference electrode; and a platinum wire as an auxiliary electrode [37]. Potential was applied to the working electrode at a constant rate (100 mV/s) receiving cyclic voltammogram. Cyclic voltammogram was composed of two parameters: the peak potential (Ep(a)), which reflects the ability to donate electrons, and the anodic current (AC), which correlates with the concentrations of these compounds [38]. Reducing power was determined from the cyclic voltammogram. The working electrode was tested prior to each series of measurements, by performing a cyclic voltammogram of 1 mm potassium ferricyanide in PBS.

### 2.7. Oxygen Radical Absorbance Capacity Assay (ORAC)

ORAC assay adapted to fluorescein labeling [39] was used to determine the total antioxidant capacity of curcumin-loaded microemulsions (the final curcumin concentrations were as follows (%*w*/*w*): 0 (empty microemulsion), 0.25 (6.8 mM), 0.5 (13.9 mM), 0.75 (20.4 mM), and 1 (27.1 mM)). Analysis was performed using 2, 2′-azobis(2-amidinopropane) dihydrochloride (AAPH) as a peroxyl generator. This assay is a kinetic assay which measures the loss of fluorescein fluorescence over time due to peroxyl radical formed by AAPH, enabling evaluation of antioxidant protection. Measurements were performed on a Fluostar Galaxy plate reader (BMG, Offenburg, Germany) equilibrated at 37°C, with excitation and emission set up at 485 nm and 520 nm, respectively. Trolox was used as a calibration standard. Reagents were prepared in phosphate buffer (pH 7.4). 40 *μ*L samples were pipetted into a 96-well plate. Fluorescein was added to a final concentration of 96 nM. ORAC fluorescence was read every 2 min for 70 min. Oxidation resulting from peroxyl radical started immediately following AAPH addition. Total antioxidant capacity was calculated by measuring the area below the kinetic curve [39].

### 2.8. Quantification of Oxidant-Scavenging Abilities (OSA) by a Luminol-Dependent Chemiluminescence (LDCL) Assay

A highly sensitive luminol-dependent chemiluminescence-inducing cocktail [40] was employed to quantify the OSA of microemulsions (the final curcumin concentrations were as follows (%*w*/*w*): 0 (empty microemulsion), 0.25 (6.8 mM), 0.5 (13.9 mM), 0.75 (20.4 mM), and 1 (27.1 mM)). Briefly, the following were added into 850 *μ*L of Hanks' balanced salt solution (HBSS) (pH 7.4): 10 *μ*L of luminol (1 mM), H_2_O_2_ (100 mM), sodium selenite (IV) (2 mM), and CoCl2·6H2O (II) (1 mM). This cocktail produces an immediate wave of light due to peroxide and hydroxyl radical. Light quenching by microemulsions indicates the degree of their oxidant-scavenging ability.

Light quenching was measured as counts per minute by a Lumac 2500 Luminometer (Landgraaf, The Netherlands).

### 2.9. Quantification of Oxidant-Scavenging Abilities (OSA) by the 2-diphenylpicrylhydrazyl (DPPH) Radical Assay

Modified DPPH assay [41] was used to determine the oxidant-scavenging ability of curcumin-loaded microemulsion (the final curcumin concentrations were as follows (%*w*/*w*): 0 (empty microemulsion), 0.25 (6.8 mM), 0.5 (13.9 mM), 0.75 (20.4 mM), and 1 (27.1 mM)). 2,2-diphenylpicrylhydrazyl (DPPH) free radical was used as a probe; upon reduction, this stable, purple, free radical changed its color to a yellow diphenylpicryl hydrazine. Briefly, 10 *μ*L of microemulsions was mixed with 20 *μ*L of a DPPH solution (10 mM in absolute methanol). One minute later, 800 *μ*L of absolute methanol was added. The reaction mixtures were centrifuged at 425 ×g for 2 min, and the change in absorption at 517 nm using a Whittaker microplate reader 2001 was determined. Oxidant-scavenging ability is expressed in terms of micromole equivalents of trolox per 100 grams of sample.

### 2.10. Cell Culture

Immortalized human keratinocytes, HaCaT cells [42], were grown in Dulbecco's Modified Eagle's Medium (DMEM, Biological Industries, Beit Haemek, Israel) containing 4.5 g/L D-glucose and supplemented with 10% fetal bovine serum, 1 mM L-glutamine, 100 U/mL penicillin, and 100 U/mL streptomycin in DMEM. The cultures were maintained in an incubator at 37°C in a humidified atmosphere of 5% CO_2_. Cells were subcultured twice weekly at a 1 : 10 ratio using a trypsin-EDTA (0.05%) solution (Biological Industries, Beit Haemek, Israel) as a detaching agent.

### 2.11. Human Skin Organ Culture

Human skin was obtained with informed consent from 20- to 60-year-old healthy women, who had gone through breast or abdomen reduction. Testing was performed according to the Declaration of Helsinki and approved by the Hadassah University Hospital Ethics Committee, #0639-12-HMO. Skin was cut into pieces of approximately 0.5 × 0.5 cm and cultured, dermal side down and epidermal side up, in 35 mm diameter petri dishes containing DMEM (Dulbecco's Modified Eagle's Medium, Biological Industries, Beit Haemek, Israel) at 37°C, under 5% CO_2_. 4 *μ*L of curcumin-loaded microemulsion (the final curcumin concentration was 1%*w*/*w* (27.1 mM)) was applied to the air-exposed epidermis 24 h before irradiation as described below. The samples were incubated for another 24 h for apoptosis determination. The epidermis was separated from the dermis by 1 min heating in phosphate-buffered saline (PBS) at 56°C and apoptosis was examined.

### 2.12. Dermal Absorption of Curcumin: An Ex Vivo Model Using Human Skin Organ Culture

Microemulsion penetration was investigated using Franz-type diffusion cells (PermeGear Inc., Hellertown, PA, USA) with a diffusion area of 1 cm^2^ and an acceptor compartment of 8 mL containing fetal bovine serum and PBS (pH 7.4) (1 : 9, *v*/*v*). Skin was mounted on Franz-type diffusion cells, epidermal side up, and dermal side facing the receptor compartment. Diffusion cells were kept at 32°C. 100 *μ*L of different treatments (the final curcumin concentration was 1%*w*/*w* (27.1 mM)) was applied to the mounted skin. Following 24 h incubation, skin was removed and washed three times using a cotton cloth containing ethanol and the viable epidermis was separated from the dermis. Separation of the full epidermis from the dermis was achieved by heat shock treatment; skin was placed for 30 seconds at 55–60°C followed by 1 min at 4°C, both in PBS. Curcumin was extracted from the separated layers with DMSO. The extraction was performed by incubation in a shaker (60 ×g) until all curcumin were released (24 h). Finally, 100 *μ*L from the receptor fluids was collected. Curcumin existence in skin layers and in the acceptor compartment was determined by measuring fluorescence excitation at 485/40 nm and emission at 528/20 nm, using a BioTek microplate reader (BioTek Instruments Inc., Winooski, VT).

### 2.13. Skin Exposure to UVB Irradiation

Prior to irradiation, culture medium was removed and skin was washed with PBS to remove all traces of treatments. PBS was added to cover the dermis, and the sample was irradiated with a UVB source (VL-6.M lamp, emission spectrum 280–350 nm, emission peak 312 nm, filter size 145 × 48 mm; Vilber Lourmat, Torcy, France) at 300 mJ/cm^2^. Immediately following irradiation, PBS was replaced by human skin organ culture medium (see above) and skin was incubated for an additional 24 h.

### 2.14. Apoptosis Determination by Caspase-3 Activity Assay

The epidermis was incubated in 100 *μ*L PBS containing 2.5 *μ*M Ac-DEVD-AMC as a substrate, with 0.02% Triton X-100 and 10 mM DTT, at 37°C in a 96-well plate [43]. Fluorescence of the released coumarin derivative was measured at 390/435 nm, using a Fluostar-BMG spectrofluorometer (Offenburg, Germany). Caspase-3 activity was calculated over 40 min in a linear range from the fluorescence versus time slope. Results were normalized relative to the control group.

### 2.15. Viability Measurements through Mitochondrial Assay

Cytotoxicity of treated cell culture (HaCaT cells) was evaluated by the MTT method described elsewhere [44]. Treatments (empty microemulsion, curcumin-loaded microemulsion, and curcumin dissolved in DMSO) according to dilutions in increasing curcumin concentrations (0–3 *μ*M) were added to 24-microwell plates containing cell cultures of 30,000 cells/mL. After 24 h, cell survival was evaluated by measuring the absorbance at 540 nm, using a Whittaker microplate reader 2001. The percentage of cell survival was normalized relative to the control group.

### 2.16. Keap1-Nrf_2_-EpRE Pathway Activation

Real-time PCR of Nrf_2_ and enzyme expression after treatments (microemulsions, free curcumin, and catalase; Sigma-Aldrich, Israel) were measured in cell culture. Subconfluent cells were treated and harvested at the desired times after treatment (see below). In the case of catalase treatments, catalase (300 U/mL) was coadministered simultaneously with the other treatments. Total RNA from cell culture was extracted according to tri-reagent protocol (Sigma). Reverse transcription was performed as previously described [45]. Aliquots of cDNA culture were subjected to real-time PCR using PerfeCTa SYBR Green SuperMix, Low ROX (Quanta Biosciences Inc.), Stratagene real-time PCR machine, and oligonucleotide sets (see oligonucleotide sequence in the Supplementary Data). In all cases, the samples were normalized relative to GAPDH expression.

### 2.17. Statistical Analysis

Experiments were performed independently at least three times. For oxidant-scavenging ability assays, each experiment included three repetitions (*n* = 3). For organ culture experiments, experiments were performed with three different donors. Each independent experiment included four repetitions, with four skin pieces being processed in parallel. Data were expressed as mean ± standard errors of the mean (SEM) or standard deviation of the mean (STDEV) as specified. Statistical significance of differences was determined using one-way ANOVA, followed by Kruskal-Wallis test. The significance threshold was set at *P* < 0.05.

## 3. Results and Discussion

### 3.1. Design of Curcumin-Loaded Microemulsion

**Figure 1:**
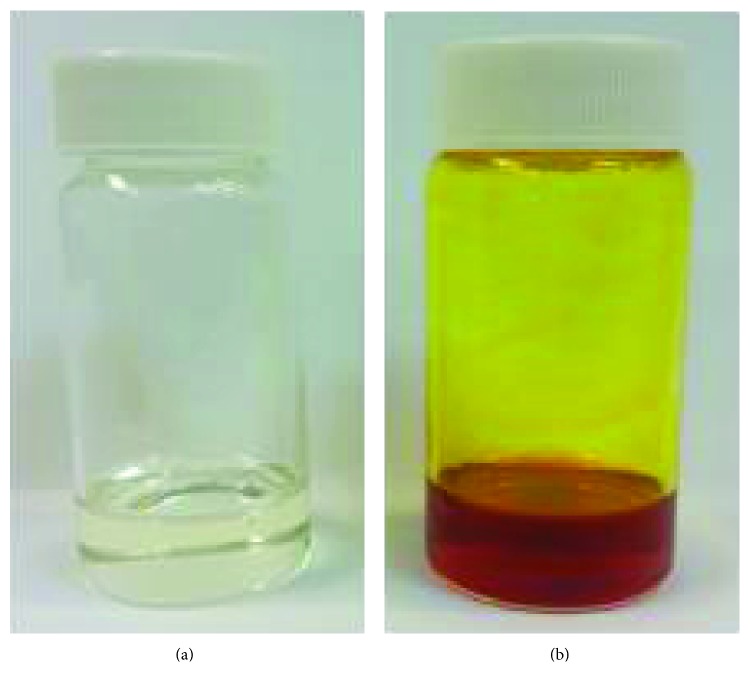
Images of (a) clear empty microemulsion and (b) curcumin-loaded microemulsion.

The usage of microemulsions for dermal delivery offers several advantages. Few mechanisms of activity were suggested in order to elucidate micreomulsion penetration ability. High solubilization capacity of the drug in the microemulsion may increase its activity towards the skin by raising the drug gradient across the skin [46] and may favor skin partition [47]. Microemulsion ingredients also have a pivotal role in the beneficial dermal delivery; surfactants and cosurfactants are often penetration enhancers resulting in the decrease in the diffusional barrier of the stratum corneum [48]. Moreover, microemulsions may have a beneficial hydration effect on the stratum corneum, influencing permeation ability [49]. Therefore, o/w microemulsions were designed. Ingredients were carefully chosen for their biocompatibility and lack of toxicity, and the usage of alcohol as a cosurfactant due to toxicity and irritancy issues [50] was denied. Nonionic surfactants were selected due to their activity as solubilizing agents and their effects on the skin barrier function [25]. The stabilization of the microemulsion was achieved using a mixture of surfactants with different HLB values.  Figure 1(a) demonstrates the ability to form an empty microemulsion formulation. Next, curcumin incorporation in the empty microemulsion formulation without disrupting its phase consistency was tested. As mentioned above, increasing curcumin's solubility would enhance its dermal delivery. Curcumin which is highly insoluble in water was solubilized in the microemulsion (Figure 1(b)). Microemulsions demonstrated stability; visual evaluation following accelerated conditions (40 ± 3°C) in darkness for 24 months revealed a transparent and isotropic behavior. Microemulsion particle size was similar to that of the freshly prepared samples with the same monomodal size distribution pattern.

### 3.2. Reduction of Cytotoxicity Using Curcumin-Loaded Microemulsions Compared to Free Curcumin in Keratinocyte

Curcumin has poor solubility in water, yet good solubility in dimethyl sulfoxide (DMSO) and chloroform [8]. Due to the low aqueous solubility of curcumin, some researchers dissolve it in base medium; however, this approach does not address the alkaline decomposition of curcumin: degradation products including ferulic acid and feruloylmethane [8]. Therefore, through all of this study, curcumin dissolved in DMSO was used as a control. DMSO curcumin solutions are termed in the following as free curcumin.

**Figure 2:**
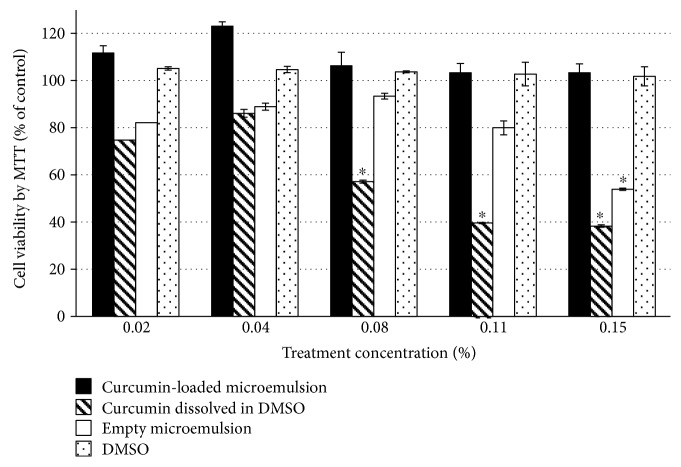
HaCaT cell viability as measured by MTT (in percentage) after 24 h treatment. Cell viability was expressed as the percentage of the untreated control (dashed line) as the function of treatment concentration. Average values are presented in the figure with standard deviation of the mean (^∗^*P* < 0.05).

Cytotoxicity of an empty microemulsion, curcumin-loaded microemulsion, and free curcumin in DMSO in immortal human keratinocyte cells (HaCaT) was measured by the MTT assay.  Figure 2 shows cell viability (%) following 24 h treatment. Axis *x* represents the treatment concentration in the cell culture (%). As can be seen, the viability of HaCaT cells exposed to an empty microemulsion at a concentration of 0.11% (*v*/*v*) or less was greater than 80%. Introduction of cells into a microemulsion at a concentration of 0.15% (*v*/*v*) significantly reduced cell viability (~50%). This decline in cell viability could be derived from the surfactants composing the microemulsion. It has been shown that all nonionic surfactants are capable of causing cell damage due to destruction of the cell membrane and its solubilization, in a concentration-dependent manner [51]. In particular, it has been shown that Tween 80 can cause cellular damage [52]. Consistent with our results (see above), it has also been shown that Tween 80 used at a concentration above 0.03% (*v*/*v*) reduced cell viability [52]. However, incorporation of curcumin into the microemulsion mitigated this cytotoxicity resulting in cell survival. Introducing free curcumin (curcumin dissolved in DMSO) demonstrates cytotoxicity at a concentration of 0.08% (*v*/*v*), indicating the curcumin-loaded microemulsion's preference in terms of cytotoxicity.

### 3.3. Structural Investigation

One of the main challenges in incorporating curcumin into a microemulsion is avoiding the interruption of microemulsion structure [53]. Cryo-TEM micrographs of the empty microemulsion and curcumin-loaded microemulsions are presented in the Supplementary Data (Figure S2). Both microemulsions demonstrate a level of order of the closely packed droplets with a diameter of about 10 nm. The dimension of microemulsion's spherical droplet was further evaluated using dynamic light scattering (DLS) measurements. As can be seen in Table 2, there is no significant difference between the size of the droplets in the empty microemulsion and that in the curcumin-loaded microemulsion. Additional nanostructure information was obtained using small-angle X-ray scattering. SAXS plots are presented in the Supplementary Data (Figures S3), demonstrating a broad peak at q ≈ 0.07 A^−1^, which corresponds to a structure with a dimension of about 9 nm according to Bragg's law, in agreement with DLS measurements and cryo-TEM images. Further analysis was done by fitting the core and shell model, which is frequently used to describe micelles and microemulsions [53]. In the case of oil-in-water microemulsions, the core is the hydrophobic component and the surfactants comprise the shell.  Table 3 reveals the best-fit parameters for the core and shell model (see Supplementary Data, Eq. 1-2), with 95% confidence bounds of the fit. The oil component (IPM) in the microemulsion has a strong influence on the microemulsion's formation and stability (data is not shown). Thus, density of the core was calculated from the oil properties (IPM) and kept constant. The shell in this model is composed from the surfactant mixture. The incorporation of curcumin into the microemulsion might affect the shell density of the microemulsion. Data in Table 3 demonstrate no significant difference in the core and shell radius between the empty microemulsion and the microemulsion containing curcumin. However, the shell density of the curcumin-loaded microemulsion is higher than the shell density of the empty microemulsion, hinting at curcumin's location in the microemulsion.

The complete model used in this study was the core and shell, with a normal size distribution of the core. Small deviations of the model from the experimental data could originate from the droplet not being ideally spherical or from the nature of the shell, which is not constant in density due to radial concentration gradients.

Curcumin's location inside the microemulsion and its interaction with the other ingredients seem to be of major importance since its mobility in the vehicle can be affected and may influence its delivery [54]. Therefore, the location of curcumin within the microemulsion was examined using the fluorescent probe method. This method can detect the microenvironment near a substance and is commonly used for revealing phase changes and structure of microemulsions and micelles [35]. Curcumin's emission properties highly depend on its specific microenvironment (e.g., polar and nonpolar solvents) [8, 36]. Therefore, curcumin can be used as a probe and directly monitor the polarity of its surroundings instead of using a probe, pointing out its site in the microemulsion. The fluorescence curves of curcumin in different solvents (background of the corresponding solvent was subtracted) are presented in the Supplementary Data (Figure S4). The wavelength of the peak is dependent on the solvent; the peak in DDW (524 nm) shifts in IPM (463-464 nm). The peak of curcumin in the microemulsion is at a wavelength of 509 nm, similar to the peak of curcumin in Tween 80 (504 nm), suggesting that the microenvironment of curcumin is alike in both the microemulsion and Tween 80 and that curcumin is located in the Tween 80 layer of the droplets. This is consistent with other studies that show that the drug is in the interface of microemulsion [53]. In addition, this data are also in agreement with SAXS data presented in Table 3 supporting curcumin's location in the surfactant shell.

### 3.4. Curcumin-Loaded Microemulsions Maintain Oxidant-Scavenging Ability In Vitro

**Figure 3:**
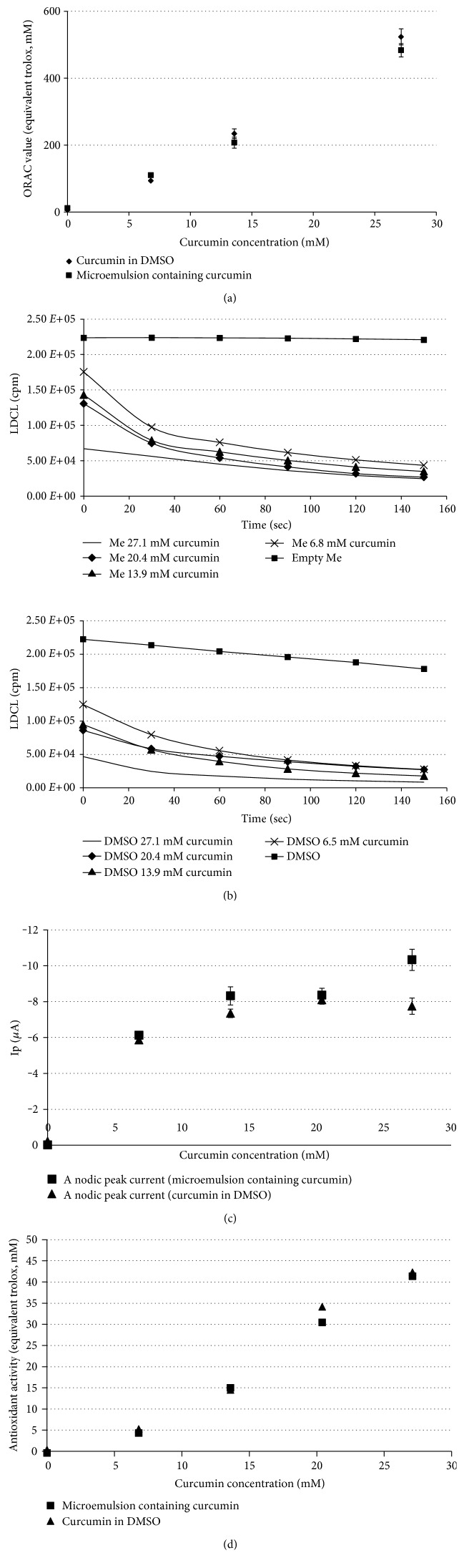
(a) ORAC value (equivalent trolox, mM) versus curcumin concentration for (■) curcumin-loaded microemulsion and (♦) curcumin dissolved in DMSO, (b) LDCL generated by “cocktail,” for curcumin-loaded microemulsion (b) and curcumin dissolved in DMSO (d) containing in increasing concentration (-) 27.1 mM curcumin, (♦) 20.4 mM curcumin, (▲) 13.9 mM curcumin, (×) 6.8 mM curcumin, and (■) microemulsion without curcumin (microemulsion and DMSO, respectively). Statistical analysis indicated significantly higher antioxidant activity indicated by a decrease in LDCL (*P* < 0.01). (c) Typical anodic peak as measured by cyclic voltammetry for (♦) curcumin dissolved in DMSO (1st oxidation potential, 407 mV) and (■) curcumin-loaded microemulsion (1st oxidation potential, 473 mV) in increasing curcumin concentrations. (e) Antioxidant activity of (♦) curcumin dissolved in DMSO and (■) curcumin-loaded microemulsion as measured by the DPPH assay and expressed in trolox equivalents.

Maintaining the oxidant-scavenging ability of curcumin loaded in microemulsions is crucial for its utilization. Therefore, antioxidant capacity was evaluated by a variety of methods on curcumin-loaded microemulsions in five increasing curcumin concentrations (%*w*/*w*): 0 (empty microemulsion), 0.25 (6.8 mM), 0.5 (13.9 mM), 0.75 (20.4 mM), and 1 (27.1 mM). Oxygen radical absorbance capacity (ORAC) assay measures the degree of inhibition of peroxyl radical-induced oxidation by the compounds of interest, expressed in trolox equivalents (*y*-axis).  Figure 3(a) demonstrates the protection of the curcumin-loaded microemulsions against the free radical. As can be seen, the microemulsion with an increased curcumin concentration demonstrates a linear trend with ORAC values expressed in trolox equivalents (ORAC = 17.451*c* − 3.9076, where *c* is the curcumin concentration in mM, coefficient of determination (*R*^2^ = 0.99)). Free curcumin (curcumin dissolved in DMSO) in increasing concentrations also demonstrates a linear trend with ORAC value (ORAC = 19.463*c* − 16.702, *R*^2^ = 0.99). Thus, curcumin-loaded microemulsions demonstrate improved protection against peroxyl radicals relative to trolox (~17.5 times more). Similar behavior is observed for free curcumin (~19.5).

The LDCL assay is based on the ability of an antioxidant agent to quench the luminescence generated by a “cocktail of oxidants.” Figure 3(b) shows that while the luminescence induced by the “cocktail” is kept steady at a high level for 2.5 min, the addition of curcumin-loaded microemulsions dramatically affects the luminescence observed. The sharp and steady decline in luminescence due to the consumption of the bulk of oxidants generated yields a curve of light emission.  Figure 3(d) demonstrates similar behavior for free curcumin (curcumin dissolved in DMSO). From calculating the area under the curve for a curcumin-loaded microemulsion and free curcumin, it can be concluded that the scavenging ability of a curcumin-loaded microemulsion is not significantly different than that of free curcumin.

Using cyclic voltammetry, the oxidation potentials of a curcumin-loaded microemulsion and free curcumin were measured. Two oxidation potentials were observed corresponding to two electron-donating centers in the curcumin molecule.  Table 4 summarizes the oxidation potentials of a curcumin-loaded microemulsion and free curcumin.  Figure 3(c) shows the anodic current at oxidation potential of 407 mV and 473 mV for free curcumin and for curcumin-loaded microemulsions, respectively. As expected, an increase in curcumin concentration resulted in an increased anodic current both for the curcumin-loaded microemulsions and for free curcumin. The anodic current drop for a curcumin-loaded microemulsion in the highest curcumin concentration might be explained by other processes involved apart from curcumin diffusion (e.g., interaction with surfactants and oils). This observation is consistent with other works [55].

The DPPH assay measures the hydrogen atom (or one electron)-donating activity and hence evaluates the antioxidant activity due to free radical scavenging. Expressed as trolox equivalents, Figure 3(e) shows that free curcumin and the curcumin-loaded microemulsions display similar antioxidant activity.

The redox assay presented here indicates that the oxygen-scavenging ability of curcumin-loaded microemulsions is similar to that of free curcumin. However, taking into consideration that curcumin dissolved in DMSO showed cytotoxicity (even in low concentrations), curcumin-loaded microemulsions may provide improved protection against free radicals without raising cytotoxicity issues. These experiments demonstrate preservation of curcumin phenolic group's activity. Thus, curcumin-loaded microemulsions can scavenge directly and potently oxygen-centered reactive intermediates.

### 3.5. Microemulsions Containing Curcumin Enhance the Activation of the Keap1-Nrf_2_-EpRE Pathway in Keratinocyte

Cellular redox homeostasis guarantees a suitable cell response to a variety of exogenous or endogenous stimuli [56]. Upon disrupting this gentle balance, reactive oxygen species which can activate proliferative and cell-survival signaling [56] can alter apoptotic pathways that may be involved in the pathogenesis of a number of skin disorders including photosensitive diseases and some types of cutaneous malignancy [15]. One of the central players involved in the redox homeostasis maintenance is the transcription factor Nrf_2_, a central key target for skin protection and cancer prevention [31]. As mentioned above, curcumin is capable of activating the Keap1-Nrf2-EpRE pathway [9]. Therefore, the effects of microemulsions on the activation of the Keap1-Nrf_2_-EpRE pathway were examined using real-time PCR. The mRNA expression of a few phase II enzymes was examined: (catalase (EC 1.11.1.6), glutathione S-transferase (EC 2.5.1.18), superoxide dismutase (EC 1.15.1.1), glutathione reductase (EC 1.8.1.7), NAD(P)H dehydrogenase [quinone] 1 (EC 1.6.5.2), and glutamate-cysteine ligase (EC 6.3.2.2) [5]. Although the relative mRNA expression of most of these enzymes was not significantly affected, the relative mRNA expression of HO-1, a known phase II enzyme, was significantly induced. HO-1 regulates the level of intracellular heme by catalyzing the oxidative degradation of heme to biliverdin, iron, and carbon monoxide, resulting in cytoprotective, antiapoptotic, and anti-inflammatory effects on various experimental models [57]. HO-1 levels are associated with the proliferating epidermis [58].

**Figure 4:**
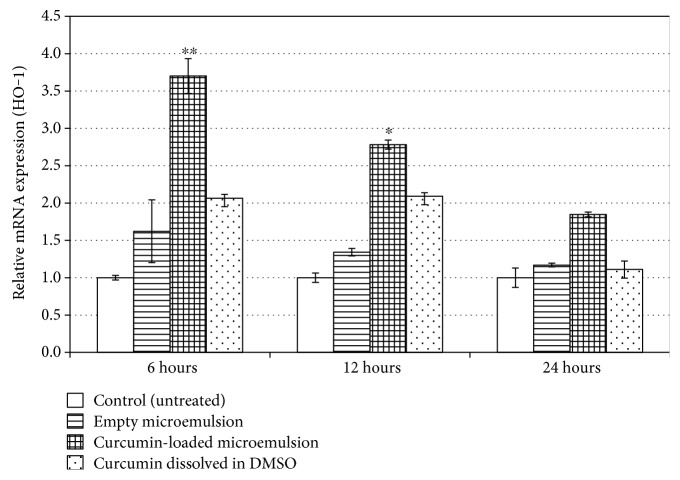
Activation of the Keap1-Nrf_2_-EpRE pathway following 6 h, 12 h, and 24 h treatments. mRNA expression determined by real-time PCR. GAPDH mRNA expression was used for normalization, and the basal mRNA normalized expression was considered 1. Average values are presented in the figure with standard deviation of the mean (^∗^*P* < 0.05, ^∗∗^*P* < 0.01).

Figure 4 demonstrates relative mRNA expression of HO-1 6, 12, and 24 h after treatments. As can be seen, the most significant relative mRNA expression increase occurs following 6 h treatment with a microemulsion containing curcumin. Empty microemulsion and free curcumin also exhibit activation of the Keap1-Nrf_2_-EpRE pathway. As can be seen, following 6 h treatment, microemulsion containing curcumin has a synergistic effect. The observation that the empty micreomulsion is capable of activating the Keap1-Nrf2-EpRE pathway can be explained by the nanodroplets composing it. It was shown that fibers and particles may activate the Keap1-Nrf2-EpRE pathway via production of reactive oxygen species [43], and we assume that the microemulsion nanodroplets operate similarly. Alternatively, the oxidation status of the micreomulsion might generate reactive oxygen species capable of activating the pathway.

Overall, our results demonstrate the advantage of curcumin-loaded microemulsions over free curcumin. Microemulsion containing curcumin enhanced the Keap1-Nrf_2_-EpRE pathway in an epidermal cell culture with a 180% increase over free curcumin. It is worth noting that tert-butylhydroquinone (tBHQ), a synthetic electrophile known for its ability in activating the Keap1-Nrf2-EpRE pathway in epidermal cell culture [59], induced the relative mRNA expression of HO-1 following 6 h treatment (50 *μ*M) similar to the curcumin-loaded microemulsions (3.5 ± 0.7 fold change). Treatment with DMSO had no effect.

### 3.6. Microemulsion Containing Curcumin Induced Activation of the Keap1-Nrf_2_-EpRE Pathway in Keratinocyte: Mechanism of Action

Polyphenols in general and curcumin in particular, under in vitro conditions, in the presence of oxygen and metal ions, may exhibit pro-oxidant activity [60]. Polyphenols can undergo autoxidation involving oxygen consumption generating O_2_·^−^, hydrogen peroxide (H_2_O_2_), semiquinones, and quinones [61]. Hydrogen peroxide (H_2_O_2_) production by polyphenols in culture media was well demonstrated [61]. H_2_O_2_ is an important mild oxidant capable of reacting with cysteines and therefore is capable of inducing several transcription factors involved in cell replication, regulation of metabolism, apoptosis, and necrosis [62]. It is worth noting that H_2_O_2_ is electronically neutral and can freely diffuse through cellular membranes [63].

An important question regarding the mechanism of activity by which the curcumin-loaded microemulsion operates in keratinocyte is whether curcumin's phenolic groups preserve their activity following incorporation into microemulsions and moreover whether curcumin retains its pro-oxidative activity. It has been shown that curcumin generated extracellular H_2_O_2_ in cell growth medium during autoxidation [60]. Therefore, it can be speculated that activation of the Keap1-NRF_2_-EpRE system is partially mediated by extracellular H_2_O_2_ production by curcumin [9]. In order to test this hypothesis, microemulsions and free curcumin were applied to keratinocyte in the presence of the enzyme catalase. H_2_O_2_ is decomposed by catalase to water and oxygen [60].

**Figure 5:**
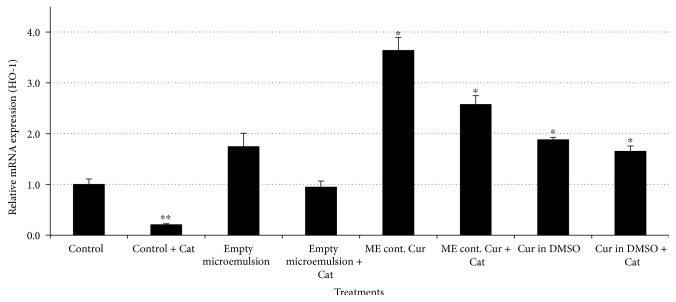
Activation of the Keap1-Nrf_2_-EpRE pathway following treatment with microemulsions (empty ME or ME containing curcumin) and free curcumin in the absence or presence of catalase (Cat, 300 U/mL). mRNA expression determined by real-time PCR. GAPDH mRNA expression was used for normalization, and the basal mRNA normalized expression was considered 1. Average values are presented in the figure with standard deviation of the mean (^∗^*P* < 0.05, ^∗∗^*P* < 0.01).

Therefore, H_2_O_2_ involvement is expected to be abrogated following catalase addition. As can be seen in Figure 5, introduction of catalase (300 U/mL) to microemulsions decreased the relative mRNA expression of HO-1 indicating lower activation of the Keap1-Nrf_2_-EpRE pathway. Catalase addition to the empty microemulsion decreased the relative mRNA expression of HO-1 from ~1.75-fold to ~0.95-fold, similar to that to the control group. This decrease demonstrated H_2_O_2_ production by the microemulsion and involvement in activation of the Keap1-Nrf_2_-EpRE pathway under experimental conditions. Curcumin-loaded microemulsions increased the relative mRNA expression of HO-1 by ~3.65-fold. As can be seen, following catalase addition, the induction was lowered to ~2.57-fold indicating that ~30% of this microemulsion's activity resulted from H_2_O_2_ involvement and the other ~70% is related to microemulsion penetration ability and curcumin's pro-oxidative activity.

It can be speculated that a few factors contribute to HO-1 induction: microemulsion's skin penetration, H_2_O_2_ involvement in curcumin phenolic groups, and the pro-oxidant activity of curcumin. Although we realize that this summation is not perfectly accurate, it is interesting to evaluate and quantify the significance of each of these contributing factors. Therefore, an assumption that the different contributions are additives was made and a rough estimation was obtained. Free curcumin treatment induced relative mRNA expression of HO-1 to increase by ~1.9-fold; following catalase addition, the induction decreased into ~1.65-fold, indicating ~12.2% H_2_O_2_ involvement and ~87.8% curcumin pro-oxidant activity. By comparing the free curcumin following catalase addition and the microemulsion containing curcumin following catalase addition, H_2_O_2_ extracellular involvement is eliminated and emphasizes the microemulsion's contribution to the means of penetration. Both treatments resulted in the induction of relative mRNA expression of HO-1 by ~1.65- and ~2.57-fold, respectively, caused by the penetration ability and curcumin's pro-oxidative activity. Since curcumin's pro-oxidative activity is exactly the same, it can be concluded that the penetration ability of the microemulsion in comparison to that in DMSO is higher by ~156% in keratinocyte. Another interesting result is the sharp decrease in relative mRNA expression of HO-1 in the control group with catalase addition, indicating H_2_O_2_ involvement in the basal state. It was shown that reactive oxygen species is formed in the cellular medium [64]; catalase addition can deplete reactive oxygen species production and therefore decrease phase II detoxification enzyme expression.

### 3.7. Evaluation of Dermal Absorption of Curcumin-Loaded Microemulsions in an Ex Vivo Model Using Human Skin Organ Culture

The skin constitutes a barrier between the body and the environment [15]. It preserves homeostasis by avoiding water loss via evaporation and protects against the environment by preventing penetration of exogenous substances [15]. Skin layers which are continuously renewed enable efficient protection against the penetration of external substances, especially thanks to the stratum corneum [65]. The outermost stratum corneum layer, despite its thickness of only 15–20 *μ*m [25], regulates the barrier properties of the skin by regulating the fluxes of chemicals and water between the environment and the organism [66]. Moreover, the hermetic barrier of the stratum corneum makes topical application challenging in spite of the large available surface area, relative low enzymatic degradation, and long application time [67].

**Figure 6:**
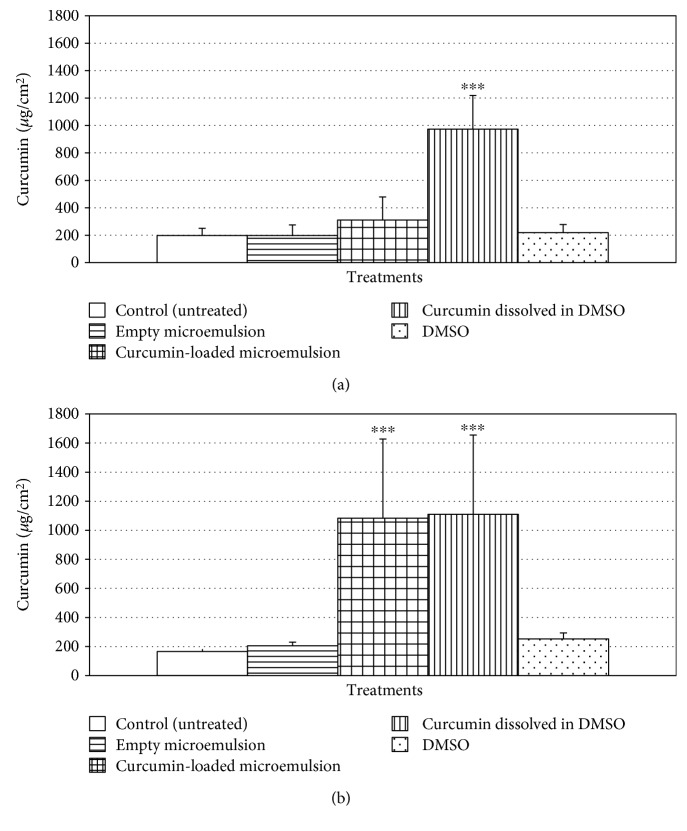
Dermal absorption evaluation of curcumin-loaded microemulsion in (a) the human skin epidermis and in (b) human skin dermis. Average values are presented in the figure with standard deviation of the mean (^∗∗∗^*P* < 0.001).

A prerequisite for the success of a dermatological drug, primarily, is its ability to penetrate through or into the skin in sufficient quantities to achieve the desired effect. A curcumin-loaded microemulsion was applied to human skin organ culture in Franz-type diffusion cells in order to perform and evaluate microemulsion's penetration ability. Curcumin was analyzed separately in the epidermis and dermis.  Figure 6 demonstrates extracted curcumin (*μ*g/cm^2^) from the epidermis (Figure 6(a) and dermis (Figure 6(b)) of human skin following topical applications. As can be seen in Figure 6(a), a significant elevation in curcumin compared to the control group (untreated skin) was observed only for application of free curcumin. However, Figure 6(b), which demonstrates curcumin's quantity in the human skin dermis, reveals that curcumin-loaded microemulsions and free curcumin (curcumin dissolved in DMSO) both penetrated the dermis by a significantly similar and elevated quantity compared to the control group (untreated skin). The observation that free curcumin was found in the epidermis is consistent with DMSO's skin adsorption enhancement properties [68]. DMSO, a polar and aprotic molecule, is one of the most efficient transdermal delivery agents [69]. However, due to its side effects (including erythema, scaling, and contact urticaria) and its potential toxicity, DMSO is rarely used as a transdermal delivery agent [69]. The ability of microemulsions to penetrate skin may be attributed to the use of penetration enhancers in the formulation, for example, isopropyl myristate, Tween 80, and Span 20 [25]. The observation that curcumin-loaded microemulsions penetrated the skin and reached the dermis in a similar quantity as free curcumin without any cytotoxicity highlights microemulsion superiority. It is worth mentioning that a similar level of fluorescence was observed in the untreated skin (control group) and in the empty microemulsion and DMSO treatments, which can be explicated by the basal levels of skin autofluorescence [44].

### 3.8. Reduction of UVB Cytotoxicity Using Curcumin-Loaded Microemulsions in Human Skin Organ Culture

**Figure 7:**
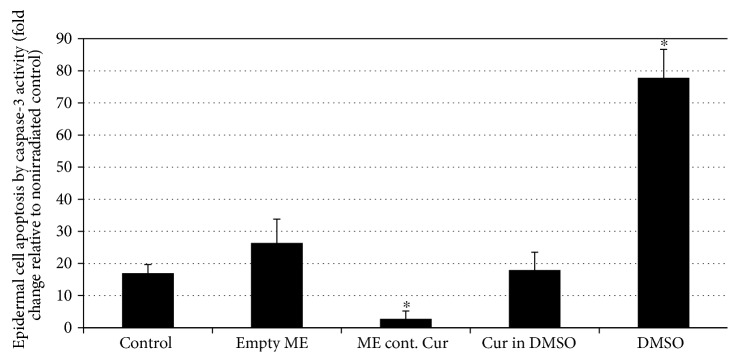
Organ cultures were treated with microemulsions for 24 h and then irradiated with UVB at 300 mJ/cm^2^, and cell apoptosis was evaluated by caspase-3 activity assay 24 h after irradiation. Data were normalized on the basis of untreated (control), nonirradiated skin (equal to 1). Average values are presented in the figure with standard deviation of the mean (^∗^*P* < 0.05).

Skin exposure to environmental stressors (e.g., UVB) may cause injury to epidermal cells through enhanced production of reactive oxygen species, thus leading to a variety of skin pathologies [15]. One of the approaches that was suggested to enable skin protection was the use of various nontoxic antioxidants which displayed efficacy in cell culture systems and animal models [15]. However, absolute efficacy in humans was not well demonstrated [15]. Topical application of Keap1-Nrf_2_-EpRE-inducing agents may present a protective strategy to reduce UVB-induced skin injury. Indeed, it has been shown that UVB-induced damage to skin cells can be efficiently limited by Keap1-Nrf_2_-EpRE-inducing agents [6]. A curcumin-loaded microemulsion was applied to human skin organ culture in order to perform and evaluate the microemulsion's ability to impede UVB-induced cell toxicity in the epidermis via Keap1-Nrf_2_-EpRE activation. Elevated HO-1 levels following 24 h incubation with the curcumin-loaded microemulsion were observed using immunohistochemical staining (indication for Keap1-Nrf_2_-EpRE pathway activation) as presented in the Supplementary Data (Figure S5). Following treatment and 24 h incubation, skin was irradiated and apoptosis was then monitored by caspase-3 activity assay. UVB irradiation caused a ~17-fold increase in caspase-3 activity indicating an increase in epidermal cell apoptosis. On the other hand, the prior application of a curcumin-loaded microemulsion reversed this trend, with only ~2.7-fold increase in caspase-3 activity in the same conditions (Figure 7). The previous application of free curcumin or an empty microemulsion did not affect caspase-3 activity significantly. However, DMSO, as expected, increased capsase-3 activity by ~78-fold. These results demonstrate an intense effect of a curcumin-loaded microemulsion to restrain UV-induced cytotoxicity in epidermal cells.

It is worth noting that curcumin's preventive effect against UVB-induced damage in skin might also be the consequence of molecular events such as downregulation of cell proliferative controls, involving thymine dimer, apoptosis, transcription factor NF-*κ*B, and inflammatory responses or upregulation of p53, and these different contributions need to be further revealed [70].

## 4. Conclusions

The work presented in this study supports the usage of curcumin-loaded microemulsions for treating oxidative stress-related conditions in skin. The incorporation of curcumin in a microemulsion, from a structural point of view, resulted in a stable nanometric-size microemulsion composed of core and shell droplets. Curcumin-loaded microemulsions maintained curcumin's activity as a reactive oxygen species scavenger. Moreover, curcumin-loaded microemulsions enabled an efficient Keap1-Nrf_2_-EpRE pathway activation.

Curcumin-loaded microemulsions promoted a powerful effect on the reduction of UV-induced cytotoxicity in epidermal cells. This work provided insights regarding the mechanism of activity in which curcumin-loaded microemulsions operate and thus supports our suggested strategy for ameliorating skin injuries and damages.

## Supplementary Material

Figure S1: chemical structure of curcumin. Figure S2: Cryo-TEM images of (A) empty microemulsion (B) curcumin-loaded microemulsion. (C) Empty microemulsion diluted 1:10 in DDW and (D) curcumin-loaded microemulsion diluted 1:10 in DDW. Figure S3: small angle X-ray scattering profiles, ln (Intensity) versus q. of (A) empty microemulsion diluted 1:10 in DDW (×). Lines were calculated from the core and shell model (eq. 1-2) with the best-fit parameters summarized in Table 2. Small angle X-ray scattering profiles, ln (Intensity) versus q, of (B) curcumin-loaded microemulsion diluted 1:10 in DDW (×). Lines were calculated from the core and shell model (Supplementary Data, eq. 1-2) with the best-fit parameters summarized in Table 3. Figure S4: Fluorescence of 0.07 mg ml-1 (1.9 µM) curcumin in water (-), IPM (◊), Tween 80 (□) or microemulsion, diluted with DDW to the desired concentration (▲). Figure S5: Paraffin-embedded skin explants were processed for immunohistochemistry by incubation with specific antibody against Heme- oxygenase-1 (HO-1; Abcam, Ab 13248, Cambridge, UK) and the appropriate secondary antibody.

## Figures and Tables

**Table 1:** Curcumin's role in treating various skin pathologies and disorders and the interconnectedness with Nrf2 [8–14, 16–19].

Skin pathology/disorder	Effect of curcumin treatment	Nrf2 involvement
Inflammatory diseases (e.g., psoriasis, atopic dermatitis, contact dermatitis, acne, rosacea, and erythroderma)	Inflammatory reduction via (1) Inhibition of NF-*κ*B transcription factor and reducing the production of TNF-*α*, IL-1, and interferon-*γ* (2) Scavenging reactive oxygen species (3) Modulating production of antioxidant enzymes	+

Scleroderma	Antifibrotic effect via suppressing TGF-*β*	+

Vitiligo vulgaris	Protection against disease progression via (1) An increase in MAPK/ERK phosphorylation and inhibition of apoptosis (2) An increase in total antioxidant capacity and a decrease in intracellular reactive oxygen species generation (3) Improving mitochondrial activity	+

Wound healing	Enhancing effective wound healing in three stages (a) Inflammation (see above) (b) Proliferation (1) Enhancing fibroblast migration, granulation tissue formation, collagen deposition, and re-epithelialization (2) Apoptosis in the early stage of wound healing resulting in removal of nondesirable inflammatory cells from the wound site (c) Remodeling (1) Enhancing wound closure via the production of TGF-*β*1 and fibronectin resulting in increased migration and proliferation of fibroblasts	+

Aging	Delay the aging process via induction of Keap1-Nrf2-EpRE and phosphatidylinositol 3-kinase/Akt pathways	+

Carcinogenesis	Anticarcinogenic activity in different stages of cancer (a) Transformation of normal cells into tumor cells: curcumin inhibits NF-*κ*B and its target genes like COX-2 and *cyclin* D1 and induces apoptosis via activation of caspase-3, caspase-8, and Fas receptor (b) Tumor growth and progression: curcumin inhibits mTOR signaling resulting in blocking of tumor progression (c) Tumor promotion: curcumin inhibits 12-o-tetradecanoylphorbol- (TPA-) induced tumor promotion and TPA-induced tumor markers via modulation of transmembrane signal transduction via protein kinase	+

**Table 2:** Diameters (averaged by volume) of empty microemulsion and microemulsion containing curcumin, as measured by dynamic light scattering. Standard deviation is specified (*N* = 6).

	Diameter (nm)
Empty microemulsion	6.75 ± 2.9
Microemulsion containing curcumin	9.33 ± 3.6

**Table 3:** Best-fit parameters for the core and shell model (Eq. 1-2), with 95% confidence bounds of the fit. Rc is the radius of the core, Rs is the radius of the droplet, and *σ* is the standard deviation of Rc (*N* = 4).

Parameter	Empty microemulsion	Microemulsion containing curcumin
Rc (nm)	3.85 ± 0.04	3.87 ± 0.07
Rs (nm)	5.39 ± 0.07	5.17 ± 0.04
Shell density (el/nm^3^)	36.34 ± 1.24	48.19 ± 2.08
*σ*	0.57 ± 0.02	0.61 ± 0.02

**Table 4:** Oxidation potential of microemulsion containing curcumin and curcumin in DMSO is measured by cyclic voltammetry.

	1st oxidation potential (mV)	2nd oxidation potential (mV)
Microemulsion containing curcumin	473	701
Curcumin dissolved in DMSO	407	702

## Abbreviations

ME: Microemulsion
SOD1: Superoxide dismutase
Nrf2: Nuclear factor (erythroid-derived 2)-like 2, NF-E2-related factor
EpRE: Electrophile response element
HO-1: Heme oxygenase-1 or haem oxygenase-1
GAPDH: Glyceraldehyde 3-phosphate dehydrogenase
NQO1: NAD(P)H dehydrogenase [quinone] 1
ROS: Reactive oxygen species
tBHQ: tert-Butylhydroquinone.
