# Supplementary material for: Curcumin Protects Skin against UVB-Induced Cytotoxicity via the Keap1-Nrf2 Pathway: The Use of a Microemulsion Delivery System
Source: Oxid Med Cell Longev. 2017 Jul 5;2017:5205471. doi: 10.1155/2017/5205471 (PMC5516744; doi:10.1155/2017/5205471)

**Supplementary Data**


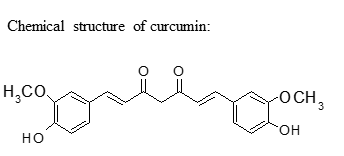


Figure S1: chemical structure of curcumin.


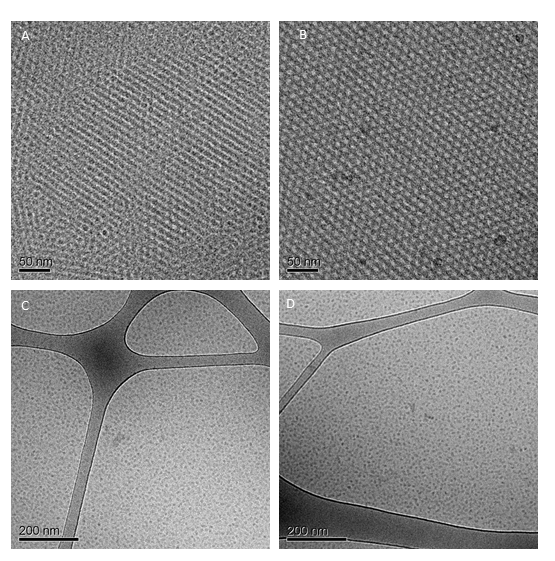


Figure S2: Cryo-TEM images of (A) empty microemulsion (B) curcumin-loaded microemulsion. (C) Empty microemulsion diluted 1:10 in DDW and (D) curcumin-loaded microemulsion diluted 1:10 in DDW.


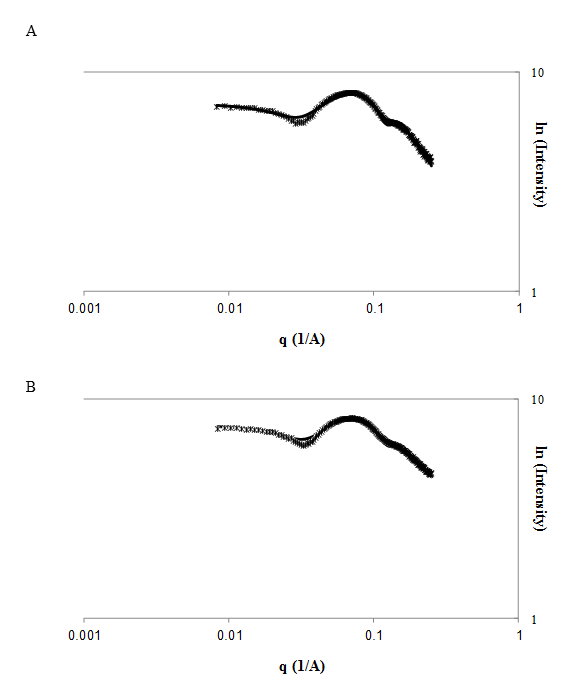


**Figure S3:** small angle X-ray scattering profiles, ln (Intensity) versus q. of (A) empty microemulsion diluted 1:10 in DDW (×). Lines were calculated from the core and shell model (eq. 1-2) with the best-fit parameters summarized in Table 2. Small angle X-ray scattering profiles, ln (Intensity) versus q, of (B) curcumin-loaded microemulsion diluted 1:10 in DDW (×). Lines were calculated from the core and shell model (Supplementary Data, eq. 1-2) with the best-fit parameters summarized in Table 3.

**Eq. 1-2:**


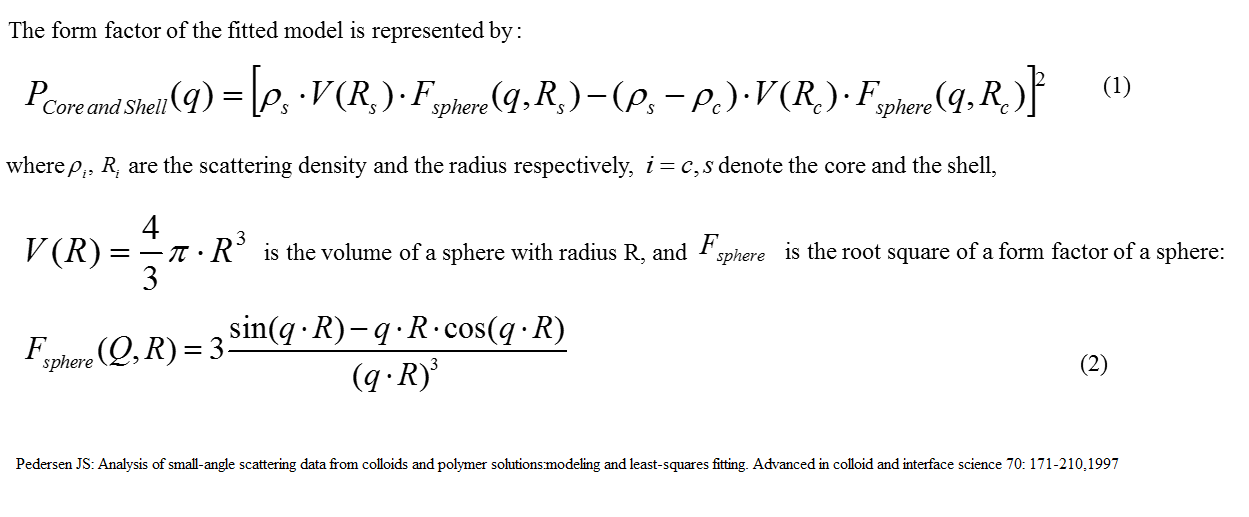


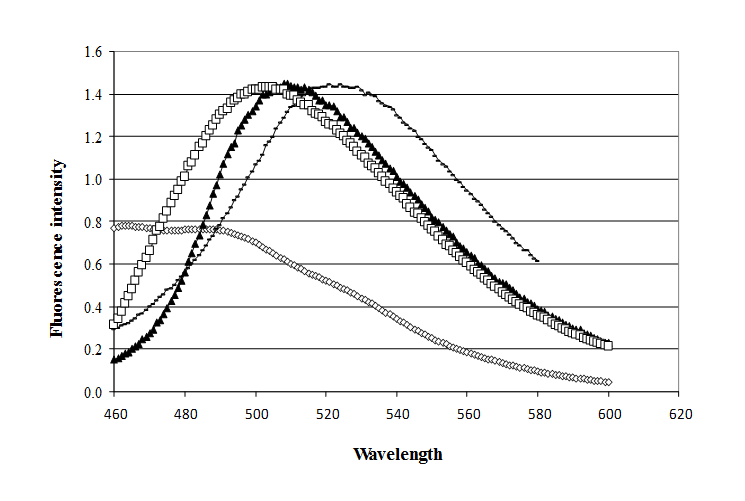


**Figure S4:** Fluorescence of 0.07 mg ml^-1^ (1.9 µM) curcumin in water (**-**), IPM (◊), Tween 80 (🞎) or microemulsion, diluted with DDW to the desired concentration (▲).


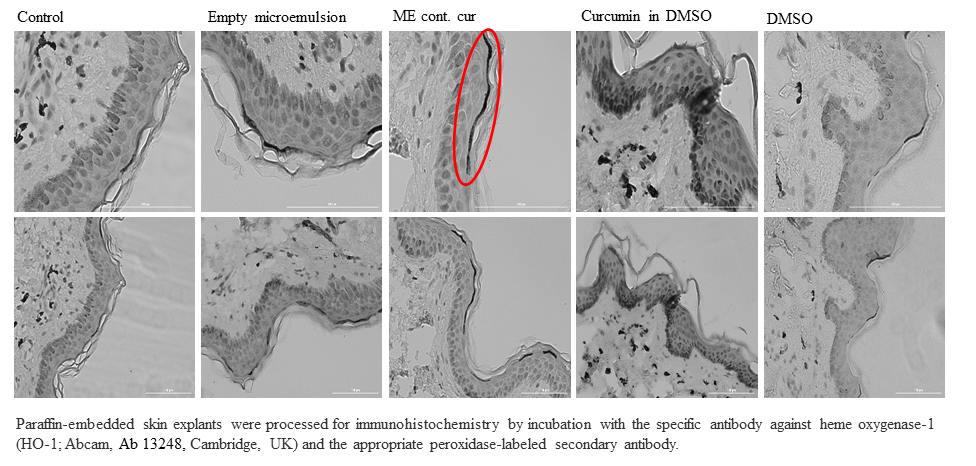


**Figure S5:** Paraffin-embedded skin explants were processed for immunohistochemistry by incubation with specific antibody against Heme- oxygenase-1 (HO-1; Abcam, Ab 13248, Cambridge, UK) and the appropriate secondary antibody.

**Description of oligonucleotide sequence:**


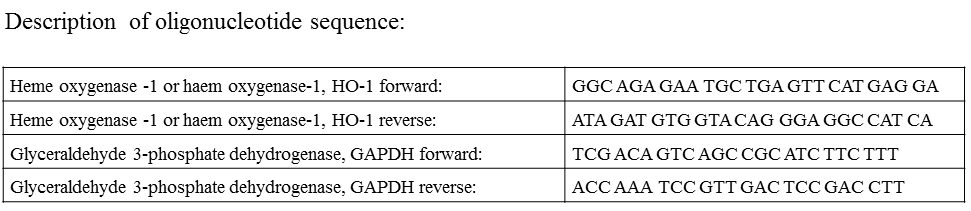

Supplement: Supplementary file 1 — Figure S1: chemical structure of curcumin. Figure S2: Cryo-TEM images of (A) empty microemulsion (B) curcumin-loaded microemulsion. (C) Empty microemulsion diluted 1:10 in DDW and (D) curcumin-loaded microemulsion diluted 1:10 in DDW. Figure S3: small angle X-ray scattering profiles, ln (Intensity) versus q. of (A) empty microemulsion diluted 1:10 in DDW (×). Lines were calculated from the core and shell model (eq. 1-2) with the best-fit parameters summarized in Table 2. Small angle X-ray scattering profiles, ln (Intensity) versus q, of (B) curcumin-loaded microemulsion diluted 1:10 in DDW (×). Lines were calculated from the core and shell model (Supplementary Data, eq. 1-2) with the best-fit parameters summarized in Table 3. Figure S4: Fluorescence of 0.07 mg ml-1 (1.9 µM) curcumin in water (-), IPM (◊), Tween 80 (□) or microemulsion, diluted with DDW to the desired concentration (▲). Figure S5: Paraffin-embedded skin explants were processed for immunohistochemistry by incubation with specific antibody against Heme- oxygenase-1 (HO-1; Abcam, Ab 13248, Cambridge, UK) and the appropriate secondary antibody. [file 5205471.f1.doc]
